# Ventilation-Induced Increases in EGFR Ligand mRNA Are Not Altered by Intra-Amniotic LPS or Ureaplasma in Preterm Lambs

**DOI:** 10.1371/journal.pone.0096087

**Published:** 2014-04-30

**Authors:** Noah H. Hillman, Tate Gisslen, Graeme R. Polglase, Suhas G. Kallapur, Alan H. Jobe

**Affiliations:** 1 Division of Neonatology, Saint Louis University, Saint Louis, Missouri, United States of America; 2 Division of Pulmonary Biology, Cincinnati Children's Hospital Medical Center, University of Cincinnati, Cincinnati, Ohio, United States of America; 3 School of Women and Infants' Health, University of Western Australia Perth, WA, Australia; 4 The Ritchie Centre, Monash Institute of Medical Research, Monash University, Melbourne, VIC, Australia; Center for Human Genetics, Germany

## Abstract

Chorioamnionitis and mechanical ventilation are associated with bronchopulmonary dysplasia (BPD) in preterm infants. Mechanical ventilation at birth activates both inflammatory and acute phase responses. These responses can be partially modulated by previous exposure to intra-amniotic (IA) LPS or Ureaplasma parvum (UP). Epidermal growth factor receptor (EGFR) ligands participate in lung development, and angiotensin converting enzyme (ACE) 1 and ACE2 contribute to lung inflammation. We asked whether brief mechanical ventilation at birth altered EGFR and ACE pathways and if antenatal exposure to IA LPS or UP could modulate these effects. Ewes were exposed to IA injections of UP, LPS or saline multiple days prior to preterm delivery at 85% gestation. Lambs were either immediately euthanized or mechanically ventilated for 2 to 3 hr. IA UP and LPS cause modest changes in the EGFR ligands amphiregulin (AREG), epiregulin (EREG), heparin binding epidermal growth factor (HB-EGF), and betacellulin (BTC) mRNA expression. Mechanical ventilation greatly increased mRNA expression of AREG, EREG, and HB-EGF, with no additional increases resulting from IA LPS or UP. With ventilation AREG and EREG mRNA localized to cells in terminal airspace. EGFR mRNA also increased with mechanical ventilation. IA UP and LPS decreased ACE1 mRNA and increased ACE2 mRNA, resulting in a 4 fold change in the ACE1/ACE2 ratio. Mechanical ventilation with large tidal volumes increased both ACE1 and ACE2 expression. The alterations seen in ACE with IA exposures and EGFR pathways with mechanical ventilation may contribute to the development of BPD in preterm infants.

## Introduction

Mechanical ventilation at birth can easily injure the preterm lung and activate a systemic acute phase response [1], [2]. Although this initial lung inflammation may contribute to the development of bronchopulmonary dysplasia (BPD) in very low birth weight infants (VLBW), other molecular pathways are also activated by mechanical ventilation [1]. Many of these pathways contribute to the later stages of lung development and perhaps repair of the initial ventilation induced injury. Even small alterations in expression may contribute to the alveolar simplification seen in infants with BPD [3]. Although clinicians have tried to decrease exposure to mechanical ventilation to decrease BPD, BPD rates have not declined substantially with the introduction of less invasive mechanical ventilation [4]-[6].

The combination of antenatal fetal exposure to chorioamnionitis and post-delivery mechanical ventilation was associated with an increased risk of BPD [7]. This is an interesting paradigm because antenatal exposure to intra-amniotic (IA) E. coli lipopolysaccharide (LPS) induces lung maturation in sheep, and clinical chorioamnionitis can decrease respiratory distress syndrome in infants [7], [8]. Nonetheless, preterm infants exposed to chorioamnionitis accompanied by fetal inflammatory response have a poor response to surfactant treatment and increased BPD [9]. Exposure to IA Ureaplasma parvum (UP) causes a milder inflammatory response than LPS, less consistent lung maturation, and reduced lung injury after mechanical ventilation [10], [11]. We previously demonstrated that IA exposure to LPS or UP modulates subsequent exposures to toll-like receptor agonists [8], [12], [13]. The development of BPD is likely promoted by a combination of multiple prenatal and antenatal exposures. Understanding the molecular pathways activated during mechanical ventilation at birth in the setting of chorioamnionitis should provide information about regulatory pathways that are activated or suppressed by fetal and early neonatal exposures.

Alterations in growth factors and metabolic pathways within the lung have been clinically associated with both BPD and lung disease in children [14], [15]. Our preliminary analysis of mRNA sequencing of mechanically ventilated lambs demonstrated possible changes in two important pathways in the lung; 1) epidermal growth factor receptor (EGFR) and 2) angiotensin converting enzymes (ACE). EGFR regulates airway branching and alveolar maturation, and mutations in EGFR receptor are found in some forms of non-small cell lung cancer [16], [17]. The EGFR ligand amphiregulin (AREG) increases with mechanical ventilation [18]. EGFR can also be triggered by multiple other ligands, including epiregulin (EREG), heparin binding- epidermal growth factor (HB-EGF), and betacellulin (BTC) [17]. ACE 1 and ACE2 are enzymes produced in the lung that can modulate lung inflammation, and ACE1 gene polymorphisms may affect severity of lung diseases [19], [20]. Using tissue from previous preterm sheep models [10]–[12], we examined whether antenatal exposure to LPS or UP alters gene expression for EGFR, EGFR ligands and ACE in the lung. We further explored the effects of mechanical ventilation on expression of these genes, with or without antenatal LPS or UP exposures, in the setting of normal (7 mL/kg) and large (15 mL/kg) tidal volume ventilation.

## Methods

The investigations were approved by the Animal Ethics Committees of the University of Western Australia and Cincinnati Children's Hospital Medical Center. Lung physiology and inflammatory responses of these animals were described previously [10]–[12]. The role of chorioamnionitis on EGFR and ACE pathways was tested using animals exposed to LPS or UP compared to unventilated controls [10], [12]. The effects of mechanical ventilation were evaluated in animals with or without intra-amniotic inflammation from LPS or UP to look for additive/protective effects [11], [12]. Mechanically ventilated, ureaplasma-exposed or saline-exposed animals were used to determine if the V_T_ used during the initial 15 minutes of ventilation affected the response of the pathways [11]. The intervention groups are described below and summarized in Table 1.

**Table 1:** Groups, intra-amniotic (IA) exposures, gestational age and ventilation variables.

LPS Groups	IA	GA at	N	V_T_ 15 min	V_T_ 105 min
	Exposure	delivery		mL/kg	mL/kg
Saline Controls (a)	Saline 2 &7d	130d	5	—	—
IA LPS (a)	LPS 2d &7d	130d	5	—	—
IA LPS+Vent (a)	LPS 2& 7d	130d	5	6.8±0.2	6.7±0.6
IA Saline+Vent (a)	Saline 2 &7d	130d	5	6.6±0.2	5.8±0.4

Ureaplasma Groups	IA		N	V_T_ 15 min	V_T_ 165 min
	Exposure			mL/kg	mL/kg
Unventilated controls (b)	none	124d	7	—	—
IA Ureaplasma (b)	14d UP	124d	5	—	—
UP UVC Controls (c)	none	128d	6	—	—
IA UP+Vent 7mL/kg (c)	55d UP	128d	5	6.8±1.1	8.2±1.2
Vent 15mL/kg (c)	none	128d	5	13.2±1.8	7.7±0.7
IA UP+Vent 15mL/kg (c)	55d UP	128d	5	14.2±1.9	7.4±0.5

(a) Gisslen *et al*. Innate immunity 2013 [12], (b) Collins *et al*. AJP-Lung 2010 [10], (c) Polglase et al. Pediatr Res 2010[11]

### LPS groups

Time-mated ewes were randomly assigned to receive 2 ml ultrasound guided IA injection of 10 mg *Escherichia coli* LPS O55:B5 (Sigma-Aldrich, St. Louis, MO) or 2 ml saline at both 123 d and 128 d gestational age before operative delivery at 130 d gestation (term is 150 d) [21]. Surfactant treatments (100 mg/kg Curosurf, a gift from Chiesi Pharma, Parma, Italy) were given to normalize surfactant pools prior to ventilation [22]. The lambs were mechanically ventilated with volume guarantee targeting a tidal volume (V_T_) of 7 ml/kg (Dräger Babylog 8000+, Lübeck, Germany) for 120 minutes [12]. Lambs receiving the two doses of LPS but no ventilation (LPS group) and lambs receiving no IA injection or ventilation (UVC) were used as comparison groups.

### Ureaplasma groups

In ureaplasma exposed lambs (UP group), lambs received 2 ml saline IA containing *Ureaplasma parvum* serovar 3 (2×10^7^ CFU) at 110 d GA and were delivered at 124 d GA, immediately euthanized, and tissues collected [10]. Ewes for ventilation studies had IA UP at 55 d gestation [23]. Fetuses in both 55 d IA UP and unexposed groups were randomized at 128±1 d gestation to one of two ventilator strategies: 1) surfactant treatment and then mechanical ventilation with a V_T_ of 7 mL/kg for 3 hours or 2) mechanical ventilation with V_T_ escalating to 15 mL/kg (V_T_15) at 15 min then surfactant treatment and ventilation for 2 hr 45 min at 7 mL/kg. Non-ventilated controls (UVC) and non-ventilated UP (UP UVC) were euthanized prior to delivery and were sampled immediately.

#### Quantitative RT-PCR

mRNA was extracted from lung tissue with TRIzol (Invitrogen, USA) and DNase treated. cDNA was produced using Verso cDNA kit (Thermoscientific, UK). Custom Taqman gene primers (Applied Biosystems, USA) were designed from ovine sequences for Amphiregulin (AREG), Epiregulin (EREG), Heparin binding-epidermal growth factor (HB-EGF), Betacellulin (BTC), Epidermal growth factor receptor (EGFR), Angiotensin converting enzyme 1 (ACE 1), ACE2, Hepatic growth factor and Midkine. Quantitative RT-PCR was performed with a 7300 RT-PCR machine and software (Applied Biosystems, USA). 18S primers were used for internal loading controls, and results are reported as fold increase over mean for unventilated control animals.

#### In situ Hybridization

In situ localization of AREG and EREG mRNA used digoxigenin-labeled anti-sense and sense sheep riboprobes synthesized from cDNA templates using DIG RNA labeling kits (Roche, USA). The sections were pre-treated with paraformaldehyde, proteinase K, and hybridized overnight. Sections were formamide washed, RNase A treated, then blocked with 10% horse serum. Following incubation overnight with anti-Digoxigenin antibody (Roche, USA), the slides were developed with NBT-BCIP (Roche, USA).

#### Data Analysis

Results are shown as mean (SEM). Significance was accepted as p<0.05 using Student's t-test or Mann-Whitney non-parametric (InStat GraphPad, USA).

## Results

The lambs were similar in size, gestational age, and ventilation variables across the groups, as previously reported [10]–[12].

### EGFR Ligand and EGFR mRNA expression

**Figure 1:**
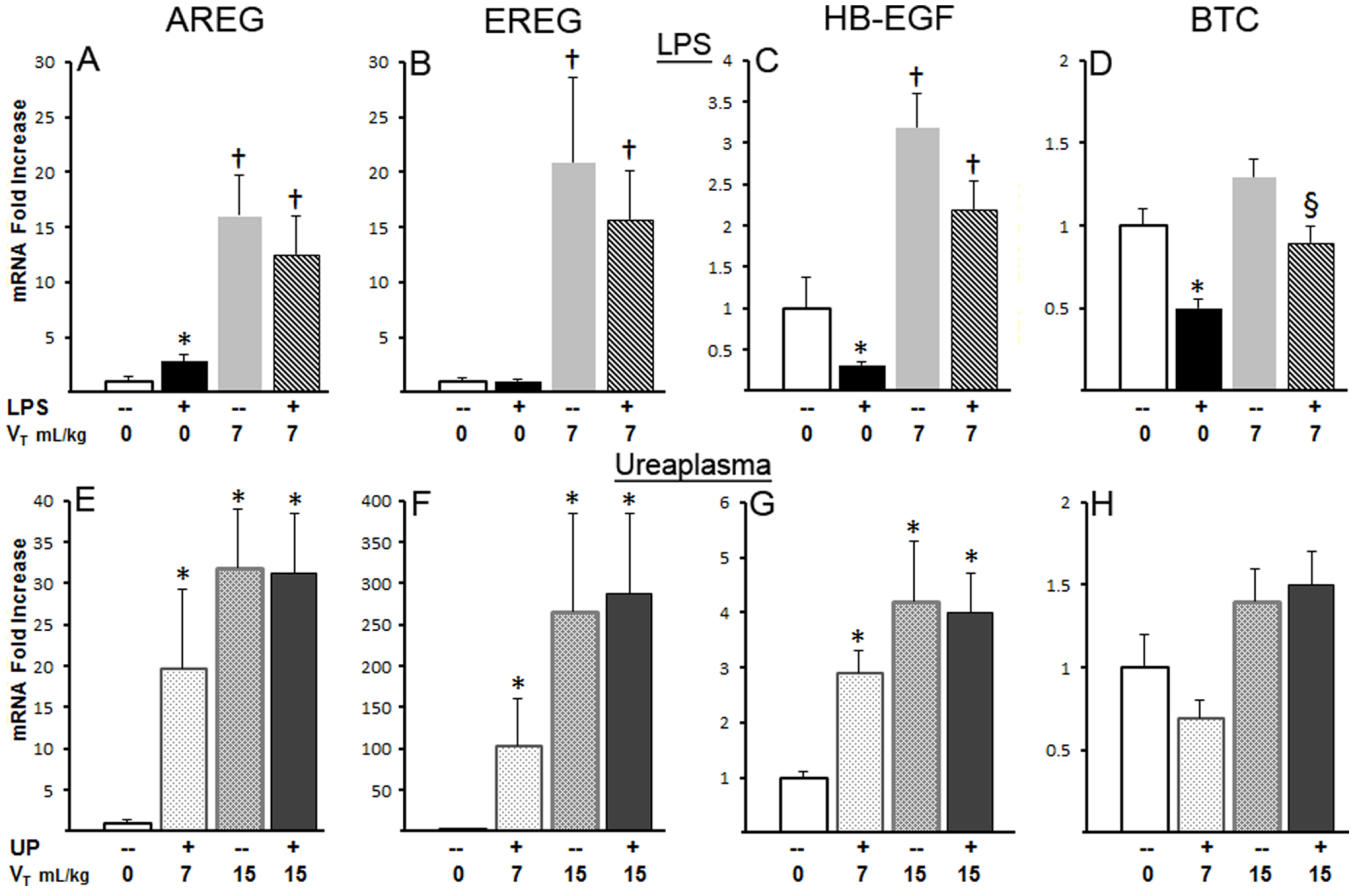
EGFR ligand mRNA response in lung to IA LPS, IA Ureaplasma, and mechanical ventilation. (**A–D**) mRNA response to IA-LPS and mechanical ventilation at 7 mL/kg (V_T_7). (A) Amphiregulin (AREG) increases with LPS exposure with further increase with V_T_7. (B) The Epiregulin (EREG) increase with V_T_7 is not altered by LPS. (C) Heparin binding epithelial growth factor (HB-EGF) decreases with LPS exposure, but increases with V_T_7. (D) Betacellulin (BTC) decreases with LPS and this decrease with LPS remains during V_T_7. (**E–H**) mRNA response to IA-UP, UP+V_T_7, mechanical ventilation at 15 mL/kg (V_T_15) and V_T_15+UP. (E) AREG and (F) EREG increase with V_T_7 and V_T_15. (G) HB-EGF increases with V_T_7 and V_T_15. (H) BTC had no change with UP or ventilation. * p<0.05 vs unventilated, unexposed controls (UVC), † p<0.05 vs UVC and IA-LPS, § p<0.05 vs UVC and V_T_7 without LPS

Intra-amniotic exposure to LPS caused a small but significant increase in AREG mRNA, and decreases in HB-EGF and BTC (p<0.05) (Figure 1A, C, D). Mechanical ventilation strongly induced mRNA for the EGFR ligands AREG, EREG, and HB-EGF, but had no effect on BTC (Figure 1). A similar magnitude increase in AREG (12 to 17 fold), EREG (15 to 50 fold), and HB-EGF (2 to 3 fold) was measured for all animals ventilated at 7 mL/kg, regardless of previous exposure to intra-amniotic LPS or UP. Animals ventilated with 15 mL/kg for 15 minutes then 7 mL/kg for 105 min had generally higher induction of AREG, EREG, HB-EGF with no effect of prior UP exposure (Figure E–G). The small decrease in BTC with LPS exposure was maintained with ventilation compared with animals not receiving IA LPS (Figure 1D). Intra-uterine exposure to Ureasplasma caused a similar small decrease in HB-EGF (Table 2).

**Table 2:** mRNA Fold increase over controls with 14 Day Ureaplasma exposure.

AREG	EREG	HB-EGF	BTC	EGFR	ACE1	ACE2	ACE1/2	Midkine	HGF
1.1±0.4	2.1±0.5	0.7±0.03*	1.0±0.2	1.0±0.1	0.7±0.03*	1.5±0.2*	2.2±0.2*	1.2±0.1	0.9±0.1

* p<0.05 vs saline controls.

**Figure 2:**
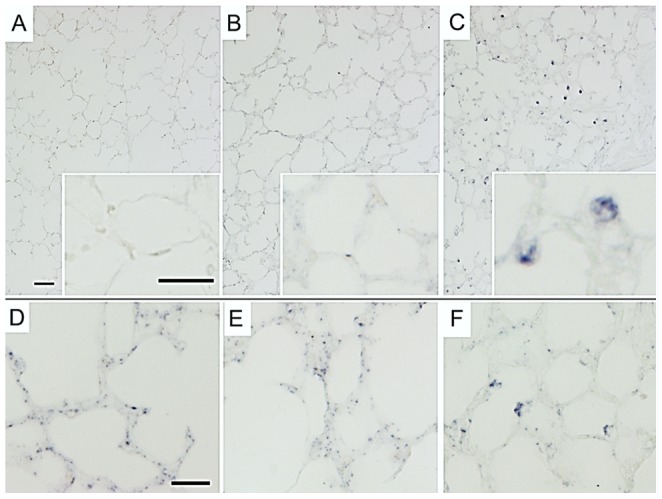
In situ localization of EREG and AREG with LPS exposure and mechanical ventilation in lung. (**A–C**) EREG mRNA was not found in (A) unventilated controls or (B) IA LPS exposed lungs, but dramatically increased in peripheral lungs of lambs receiving (C) mechanical ventilation. EREG mRNA is localized to dome-shaped cells in peripheral lung (C, Insert). (**D–F**) AREG mRNA is found scattered in the peripheral lung of (D) Unventilated controls with a mild increase with (E) IA LPS exposure. (F) Mechanical ventilation increases the mRNA signal in similar cells to EREG. Scale bar = 50 µm

In situ localization of EREG demonstrated increased mRNA signal in domed cells within the distal lung (Figure 2C, Insert). Control lambs and lambs exposed to LPS did not demonstrate in situ signal (Figure 2A,B). AREG mRNA signal is also increased in distal domed cells with ventilation (Figure 2F), and not found in similar appearing cells in either control (Figure 2D) or LPS exposed animals (Figure 2E). Cells with AREG and EREG mRNA signal did not co-stain with smooth muscle actin (data not shown), and are located in appropriate location for type II pneumocytes.

IA LPS exposure caused a 30% decrease in EGFR mRNA (p<0.05) (Table 3), whereas IA UP had no effect (Table 2). Mechanical ventilation, with or without IA LPS or UP, caused an average 1.5±0.1 fold increase in EGFR mRNA (p<0.05 in each ventilated groups vs. controls) (Table 3).

**Table 3:** EGFR and Midkine mRNA fold changes.

Groups	EGFR	Midkine
**Controls**	1.0±0.1	1.0±0.1
**LPS**	0.7±0.04*	0.9±0.1
**Vent 7 mL/kg**	1.3±0.1^†^	0.7±0.1
**LPS+Vent 7 mL/kg**	1.4±0.1^†^	0.5±0.1^†^
**Vent 15 mL/kg**	1.6±0.2*	1.1±0.1
**UP+Vent 15 mL/kg**	1.8±0.3*	0.8±0.1

*p<0.05 vs Controls; ^†^p<0.05 vs Controls and LPS alone.

### Angiotensin Converting Enzyme mRNA

**Figure 3:**
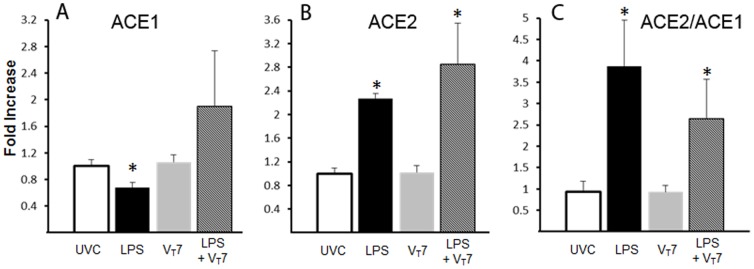
Angiotensin converting enzyme (ACE) 1 and 2 mRNA in lung with exposure to LPS, UP and mechanical ventilation. (**A–C**) mRNA values for unventilated controls (UVC), IA-LPS, and mechanical ventilation at 7 mL/kg (V_T_7). (A) ACE1 was decreased by LPS but unaffected by V_T_7. (B) ACE2 was increased by LPS and LPS+V_T_7. (C) ACE2/ACE1 mRNA ratio increased with LPS and LPS+V_T_7. * p<0.05 vs UVC.

Angiotensin converting enzyme (ACE) is produced in the lung and the balance between ACE 1 and ACE2 may contribute to lung injury or repair [19]. IA LPS exposure decreased in ACE1 mRNA (Figure 3A) and increased ACE2 mRNA (Figure 3B), resulting in a 4-fold increase in the ratio of ACE2/ACE1 (Figure 3C). 14 day exposure to UP caused a similar decrease in ACE1 mRNA and a small increase in ACE2 mRNA, which increased the ACE2/ACE1 ratio 2 fold (Table 2). Mechanical ventilation with 7 mL/kg did not alter ACE mRNA (Figure 3A–C). Ventilation with 15 mL/kg increased ACE1 mRNA (1.5±0.1 fold), ACE2 mRNA (2.3±0.2 fold), and the ratio of ACE2/ACE1 (1.6±0.3 fold) versus unventilated controls (p<0.05 for all). Midkine mRNA decreased somewhat with ventilation of LPS exposed lambs (Table 3). There were no changes in the mRNA for midkine or hepatic growth factor with 14 d UP (Table 2).

## Discussion

Chorioamnionitis and mechanical ventilation are associated with an increased risk of BPD in VLBW infants [7]. Growth factors important for lung development (EGFR and its ligands) and ACE1, ACE2 in the lung were altered by either antenatal inflammation or mechanical ventilation. We demonstrated a striking increase in the mRNA for EGFR ligands with mechanical ventilation at birth, especially with a V_T_ of 15 mL/kg, and a subsequent 1.5 fold increase in EGFR mRNA. Baseline EGFR mRNA ligands were modestly changed with antenatal exposure to LPS or UP. In contrast to the EGFR ligands, ACE1 and ACE2 were altered by antenatal exposure to LP or UP, but minimally changed by mechanical ventilation. These findings identify several molecular pathways activated at birth that may contribute to the development of BPD.

One of our primary findings is the striking up-regulation of multiple EGFR ligands during mechanical ventilation of preterm lambs. Our observation that AREG increased with mechanical ventilation is consistent with gene arrays that demonstrate increased AREG in ventilated adult mice, but not to the degree seen in these preterm lambs [18]. In isolated mouse lungs, ventilation with a large tidal volume increased AREG mRNA and protein compared to lungs receiving more moderate tidal volumes [18]. The AREG protein was increased in the peripheral lung, though exact cell type was not determined [18]. In contrast to our antenatal exposure, postnatal LPS exposure in mice further increased the mRNA values when lungs were ventilated with high tidal volumes and LPS exposure [18]. EGFR ligands can also be excreted by pulmonary cells in response to stimulation; AREG is secreted from human pulmonary epithelial cells with exposure to cigarette smoke or particulate matter [24]. Mechanical compression of human bronchial epithelial cells (NHBE cells) caused the shedding of HB-EGF into the extracellular fluid and an autocrine activation of the EGFR receptor [25]. Mechanical stretch also released HB-EGF, EGF, AREG, and BTC [26]. The trends for further increases in mRNA for EGFR ligands in lambs receiving more injurious ventilation (15 mL/kg) corresponded with our previously reported increased inflammatory markers with higher V_T_ ventilation in the preterm lung [11]. This result is consistent with *in vitro* studies of stretched type II cells, where increased duration of stretch caused larger mRNA induction [27]. Our findings and previous studies demonstrate an increase in multiple EGFR ligands and EGFR mRNA in response to mechanical ventilation, and the intensity of the response corresponds to the degree of mechanical stretch.

The production of mRNA for EGFR ligands by domed-cells in the peripheral lung with mechanical ventilation may have a large effect on the developing alveoli and could contribute to the development of BPD. EGF has an important role in lung development and cellular differentiation. EGFR deficient mice have decreased airway branching, decreased alveolarization, type II cell immaturity, and respiratory distress at birth [28]. Similarly, pregnant rats given anti-EGF antibodies deliver pups with respiratory distress and decreased lung size [29]. Fetal administration of EGF to preterm rabbits caused increased lung maturation and number of type II alveolar epithelial cells [30]. HB-EGF promotes type II epithelial differentiation with increased mRNA for surfactant proteins B and C, whereas type II cells incubated with AREG, EREG or BTC did not have increased surfactant mRNA [26]. Over-expression of EGFR can also cause injury, as EGF stimulation in type II cells can lead to alveolar tumors and EGFR gene mutations are found in about 10% of non-small cell lung tumors [31]. AREG also participates in pulmonary fibrosis by modulation of TGF-α [32], and EGFR inhibitors can decrease pulmonary fibrosis[33]. Alterations of EGF, EGFR, and TGF- α were reported in lungs from infants with BPD [34].Increased expression of AREG also contributes to the mucus cell metaplasia seen in naphthalene-lung injury [35]. In contrast, AREG given intra-peritoneally to mice decreased the lung inflammation and fibrosis caused by bleomycin [36]. It is presently unclear whether the production of EGFR ligands during mechanical ventilation is a protective or harmful response of the preterm lung.

Although we did not measure a large effect of antenatal exposure to LPS or Ureaplasma on EGFR ligand expression, IA exposure can alter pro-inflammatory pathways in fetal sheep [8], [12], [13]. Long term exposure to UP decreased the response to IA LPS, demonstrating fetal cross-tolerance to toll-like receptors [13]. Fetal sheep develop tolerance when exposed to multiple doses of E. Coli LPS [8]. IA LPS also causes a partial tolerance to IV LPS in ventilated preterm sheep [12]. Since IA LPS also matures the macrophages within the lungs and alters their response subsequent stimulation, the lack of large changes in EGFR ligands with IA LPS or Ureaplasma suggests these cells are likely not the primary source of EGFR activation [37]. The additive effects of chorioamnionitis and mechanical ventilation [7] are likely not due to changes in EGFR or ligands.

We demonstrated a decrease in ACE1 mRNA and an increase in ACE2 mRNA with exposure to intra-amniotic LPS and UP. There was little additional effect on the ACE ratio with short-term mechanical ventilation at 7 mL/kg, though larger V_T_ ventilation increased both ACE1 and ACE2 mRNA. The change in the ACE ratio from the antenatal exposure may also contribute to BPD by effects on lung inflammation. ACE1 cleaves angiotensin I to angiotensin II, which is a potent activator of a variety of receptors, and ACE2 can inactivate angiotensin II and convert it to Angiotensin 1–7. ACE1 activation causes lung inflammation by activation of NF-kB [19], and ACE enzyme activity is elevated in the airway fluid of patients with ARDS [15]. ACE1 inhibitors and angiotensin II receptor antagonists decrease lung inflammation and fibrin deposition in multiple animal models of ARDS [19], [38]. There is speculation that polymorphisms in ACE gene contribute to mortality from ARDS [20]. In small clinical studies, ACE gene polymorphisms were not associated with BPD or RDS [39], [40]. ACE2 protected the lung against injury in animal models of ARDS [41], and supplementation of recombinant ACE2 decreased lung fibrosis in rats exposed to bleomycin-induced lung injury [42]. ACE1 and ACE2 enzyme activity counter-balance each other within the lung, and the ratio of the enzymes (ACE1/ACE2) correlates with the extent of lung injury [43]. In rats exposed to intratracheal LPS 24 hours prior to ventilation, ACE1 increases and ACE2 decreases, but only in the ventilated animals [43]. Correction of the imbalance of ACE2/ACE1 activity with either Angiontesin 1-7 or blockade of ACE1 activity with losartan decreased the lung injury [43]. We found an opposite response in the ACE2/ACE1 ratio, likely due to antenatal LPS exposure in preterm lambs. The components of the renin-angiotensin system are present in the mouse lung at the pseudoglandular stage of lung development (E13.5) and angiotensin II contrtibutes to increased lung branching in lung explants [44]. ACE inhibitors given to neonatal rats cause larger airspaces, thinner alveolar septum, and lower surface tension in the BAL, suggesting a role of the angiotensin system in lung development [45]. Although modest, the two to four-fold change in the ACE2/ACE1 ratio caused by antenatal exposure to LPS or UP could contribute to in the alveolar simplification of BPD.

One of the limitations of the study is the assumption that mRNA is translated into protiens and have a physiologic response. The consistency and magnitude of mRNA increases for multiple ligands across 3 animal groups, and subsequent increase in mRNA for the receptor suggest the mRNA is translated. The mRNA increases are represented as fold increases over control values, but not as absolute values of mRNA. Although only a rough estimate of relative abundancy, the Ct values were 5 cycles earlier in HB-EGF and EGFR mRNA than for AREG and EREG, suggesting these mRNA are considerably more abundant. The increases in AREG and EREG mRNA from control baseline were similar to those for the cytokines previously reported [11], [12]. A similar 5 cycle difference in Ct is also seen between ACE1 and ACE2 mRNA, suggesting ACE1 is more prevelant in the developing lung. Interpretation of Ct values must be done with some caution, as the relative efficiency of each PCR probe may contribute to differences, thus fold change over controls was reported.

Lung and systemic inflammation are associated with both the initiation of ventilation of preterm lambs and chorioamnionitis, and likely contribute to BPD [10], [17], [40]. We now demonstrate two additional molecular pathways that add more complexity to understanding the pathogenesis of BPD. Mechanical ventilation of preterm lambs caused an increase in EGFR ligands which can influence cell differentiation, but the EGFR ligands were not modulated by LPS or UP exposure. Conversely, the ratio of ACE1 to ACE2 was altered by prenatal exposure to LPS or UP but not altered by short term mechanical ventilation. The link between chorioamnionitis and mechanical ventilation may not be an additive effect on a specific molecular pathway, but a combination of effects on multiple pathways. Understanding the additional molecular pathways activated in premature infants by mechanical ventilation may provide new therapeutic treatment for infants with BPD.

## References

[1] Hillman NH, Moss TJ, Nitsos I, Jobe AH (2012) Moderate tidal volumes and oxygen exposure during initiation of ventilation in preterm fetal sheep. Pediatr Res 72: 593–599. 23037872 10.1038/pr.2012.135 PMC4073615

[2] Hillman NH, Moss TJ, Kallapur SG, Bachurski C, Pillow JJ, et al (2007) Brief, large tidal volume ventilation initiates lung injury and a systemic response in fetal sheep. Am J Respir Crit Care Med 176: 575–581. 17641159 10.1164/rccm.200701-051OC PMC1994225

[3] Jobe AH (1999) The New BPD: An arrest of lung development. Pediatric Research 46: 641–643. 10590017 10.1203/00006450-199912000-00007

[4] Finer NN, Carlo WA, Walsh MC, Rich W, Gantz MG, et al (2010) Early CPAP versus surfactant in extremely preterm infants. N Engl J Med 362: 1970–1979. 20472939 10.1056/NEJMoa0911783 PMC3071534

[5] Morley CJ, Davis PG, Doyle LW, Brion LP, Hascoet JM, et al (2008) Nasal CPAP or intubation at birth for very preterm infants. N Engl J Med 358: 700–708. 18272893 10.1056/NEJMoa072788

[6] Fischer HS, Buhrer C (2013) Avoiding Endotracheal Ventilation to Prevent Bronchopulmonary Dysplasia: A Meta-analysis. Pediatrics 132: e1351–1360. 24144716 10.1542/peds.2013-1880

[7] Van Marter LJ, Dammann O, Allred EN, Leviton A, Pagano M, et al (2002) Chorioamnionitis, mechanical ventilation, and postnatal sepsis as modulators of chronic lung disease in preterm infants. J Pediatr 140: 171–176. 11865267 10.1067/mpd.2002.121381

[8] Kallapur SG, Jobe AH, Ball MK, Nitsos I, Moss TJ, et al (2007) Pulmonary and systemic endotoxin tolerance in preterm fetal sheep exposed to chorioamnionitis. J Immunol 179: 8491–8499. 18056396 10.4049/jimmunol.179.12.8491

[9] Been JV, Rours IG, Kornelisse RF, Jonkers F, de Krijger RR, et al. (2010) Chorioamnionitis alters the response to surfactant in preterm infants. J Pediatr 156: : 10–15 e11. 10.1016/j.jpeds.2009.07.044 19833352

[10] Collins JJ, Kallapur SG, Knox CL, Nitsos I, Polglase GR, et al (2010) Inflammation in fetal sheep from intra-amniotic injection of Ureaplasma parvum. Am J Physiol Lung Cell Mol Physiol 299: L852–860. 20935228 10.1152/ajplung.00183.2010 PMC3006269

[11] Polglase GR, Hillman NH, Pillow JJ, Nitsos I, Newnham JP, et al (2010) Ventilation-mediated injury after preterm delivery of Ureaplasma parvum colonized fetal lambs. Pediatr Res 67: 630–635. 20220549 10.1203/PDR.0b013e3181dbbd18

[12] Gisslen T, Hillman NH, Musk GC, Kemp MW, Kramer BW, et al.. (2013) Repeated exposure to intra-amniotic LPS partially protects against adverse effects of intravenous LPS in preterm lambs. Innate Immun. 10.1177/1753425913488430 23751819

[13] Kallapur SG, Kramer BW, Knox CL, Berry CA, Collins JJ, et al (2011) Chronic Fetal Exposure to Ureaplasma parvum Suppresses Innate Immune Responses in Sheep. J Immunol 187: 2688–2695. 21784974 10.4049/jimmunol.1100779 PMC3159703

[14] Been JV, Debeer A, van Iwaarden JF, Kloosterboer N, Passos VL, et al (2010) Early alterations of growth factor patterns in bronchoalveolar lavage fluid from preterm infants developing bronchopulmonary dysplasia. Pediatr Res 67: 83–89. 19770691 10.1203/PDR.0b013e3181c13276

[15] Idell S, Kueppers F, Lippmann M, Rosen H, Niederman M, et al (1987) Angiotensin converting enzyme in bronchoalveolar lavage in ARDS. Chest 91: 52–56. 3024928 10.1378/chest.91.1.52

[16] Marchetti A, Martella C, Felicioni L, Barassi F, Salvatore S, et al (2005) EGFR mutations in non-small-cell lung cancer: analysis of a large series of cases and development of a rapid and sensitive method for diagnostic screening with potential implications on pharmacologic treatment. J Clin Oncol 23: 857–865. 15681531 10.1200/JCO.2005.08.043

[17] Sibilia M, Wagner EF (1995) Strain-dependent epithelial defects in mice lacking the EGF receptor. Science 269: 234–238. 7618085 10.1126/science.7618085

[18] Dolinay T, Kaminski N, Felgendreher M, Kim HP, Reynolds P, et al (2006) Gene expression profiling of target genes in ventilator-induced lung injury. Physiol Genomics 26: 68–75. 16569776 10.1152/physiolgenomics.00110.2005

[19] Ortiz LA, Champion HC, Lasky JA, Gambelli F, Gozal E, et al (2002) Enalapril protects mice from pulmonary hypertension by inhibiting TNF-mediated activation of NF-kappaB and AP-1. Am J Physiol Lung Cell Mol Physiol 282: L1209–1221. 12003776 10.1152/ajplung.00144.2001

[20] Matsuda A, Kishi T, Jacob A, Aziz M, Wang P (2012) Association between insertion/deletion polymorphism in angiotensin-converting enzyme gene and acute lung injury/acute respiratory distress syndrome: a meta-analysis. BMC Med Genet 13: 76. 22938636 10.1186/1471-2350-13-76 PMC3459791

[21] Jobe AH, Newnham JP, Willet KE, Sly P, Ervin MG, et al (2000) Effects of antenatal endotoxin and glucocorticoids on the lungs of preterm lambs. Am J Obstet Gynecol 182: 401–408. 10694344 10.1016/s0002-9378(00)70231-6

[22] Hillman N, Kallapur SG, Pillow JJ, Polglase GR, Nitsos I, et al (2010) Inhibitors of inflammation and endogenous surfactant pool size as modulators of lung injury with initiation of ventilation in preterm sheep. Resp Research 11: 1–8. 10.1186/1465-9921-11-151 PMC2978154 21034485

[23] Moss TJ, Knox CL, Kallapur SG, Nitsos I, Theodoropoulos C, et al. (2008) Experimental amniotic fluid infection in sheep: effects of Ureaplasma parvum serovars 3 and 6 on preterm or term fetal sheep. Am J Obstet Gynecol 198: : 122 e121–128. 10.1016/j.ajog.2007.06.065 PMC2213425 18166324

[24] Blanchet S, Ramgolam K, Baulig A, Marano F, Baeza-Squiban A (2004) Fine particulate matter induces amphiregulin secretion by bronchial epithelial cells. Am J Respir Cell Mol Biol 30: 421–427. 14701705 10.1165/rcmb.2003-0281RC

[25] Tschumperlin DJ, Dai G, Maly IV, Kikuchi T, Laiho LH, et al (2004) Mechanotransduction through growth-factor shedding into the extracellular space. Nature 429: 83–86. 15103386 10.1038/nature02543 PMC5539413

[26] Wang Y, Maciejewski BS, Soto-Reyes D, Lee HS, Warburton D, et al (2009) Mechanical stretch promotes fetal type II epithelial cell differentiation via shedding of HB-EGF and TGF-alpha. J Physiol 587: 1739–1753. 19237431 10.1113/jphysiol.2008.163899 PMC2683961

[27] Yerrapureddy A, Tobias J, Margulies SS (2010) Cyclic stretch magnitude and duration affect rat alveolar epithelial gene expression. Cell Physiol Biochem 25: 113–122. 20054150 10.1159/000272056 PMC3025888

[28] Miettinen PJ, Warburton D, Bu D, Zhao JS, Berger JE, et al (1997) Impaired lung branching morphogenesis in the absence of functional EGF receptor. Dev Biol 186: 224–236. 9205141 10.1006/dbio.1997.8593

[29] Raaberg L, Nexo E, Jorgensen PE, Poulsen SS, Jakab M (1995) Fetal effects of epidermal growth factor deficiency induced in rats by autoantibodies against epidermal growth factor. Pediatr Res 37: 175–181. 7731754 10.1203/00006450-199502000-00009

[30] Catterton WZ, Escobedo MB, Sexson WR, Gray ME, Sundell HW, et al (1979) Effect of epidermal growth factor on lung maturation in fetal rabbits. Pediatr Res 13: 104–108. 311914 10.1203/00006450-197902000-00004

[31] Lin C, Song H, Huang C, Yao E, Gacayan R, et al (2012) Alveolar type II cells possess the capability of initiating lung tumor development. PLoS One 7: e53817. 23285300 10.1371/journal.pone.0053817 PMC3527621

[32] Zhou Y, Lee JY, Lee CM, Cho WK, Kang MJ, et al (2012) Amphiregulin, an epidermal growth factor receptor ligand, plays an essential role in the pathogenesis of transforming growth factor-beta-induced pulmonary fibrosis. J Biol Chem 287: 41991–42000. 23086930 10.1074/jbc.M112.356824 PMC3516745

[33] Hardie WD, Davidson C, Ikegami M, Leikauf GD, Le Cras TD, et al (2008) EGF receptor tyrosine kinase inhibitors diminish transforming growth factor-alpha-induced pulmonary fibrosis. Am J Physiol Lung Cell Mol Physiol 294: L1217–1225. 18424623 10.1152/ajplung.00020.2008

[34] Strandjord TP, Clark JG, Guralnick DE, Madtes DK (1995) Immunolocalization of transforming growth factor-alpha, epidermal growth factor (EGF), and EGF-receptor in normal and injured developing human lung. Pediatr Res 38: 851–856. 8618784 10.1203/00006450-199512000-00005

[35] Manzo ND, Foster WM, Stripp BR (2012) Amphiregulin-dependent mucous cell metaplasia in a model of nonallergic lung injury. Am J Respir Cell Mol Biol 47: 349–357. 22493011 10.1165/rcmb.2011-0257OC PMC3488692

[36] Fukumoto J, Harada C, Kawaguchi T, Suetsugu S, Maeyama T, et al (2010) Amphiregulin attenuates bleomycin-induced pneumopathy in mice. Am J Physiol Lung Cell Mol Physiol 298: L131–138. 19915156 10.1152/ajplung.90576.2008

[37] Kramer BW, Joshi SN, Moss TJ, Newnham JP, Sindelar R, et al (2007) Endotoxin-induced maturation of monocytes in preterm fetal sheep lung. Am J Physiol Lung Cell Mol Physiol 293: L345–353. 17513458 10.1152/ajplung.00003.2007

[38] Wang F, Xia ZF, Chen XL, Jia YT, Wang YJ, et al (2009) Angiotensin II type-1 receptor antagonist attenuates LPS-induced acute lung injury. Cytokine 48: 246–253. 19748795 10.1016/j.cyto.2009.08.001

[39] Ince DA, Atac FB, Ozkiraz S, Dilmen U, Gulcan H, et al (2010) The role of plasminogen activator inhibitor-1 and angiotensin-converting enzyme gene polymorphisms in bronchopulmonary dysplasia. Genet Test Mol Biomarkers 14: 643–647. 20818980 10.1089/gtmb.2010.0072 PMC2957238

[40] Satar M, Taskin E, Ozlu F, Tuli A, Ozcan K, et al (2012) Polymorphism of the angiotensin-converting enzyme gene and angiotensin-converting enzyme activity in transient tachypnea of neonate and respiratory distress syndrome. J Matern Fetal Neonatal Med 25: 1712–1715. 22339243 10.3109/14767058.2012.663017

[41] Imai Y, Kuba K, Rao S, Huan Y, Guo F, et al (2005) Angiotensin-converting enzyme 2 protects from severe acute lung failure. Nature 436: 112–116. 16001071 10.1038/nature03712 PMC7094998

[42] Rey-Parra GJ, Vadivel A, Coltan L, Hall A, Eaton F, et al (2012) Angiotensin converting enzyme 2 abrogates bleomycin-induced lung injury. J Mol Med (Berl) 90: 637–647. 22246130 10.1007/s00109-012-0859-2 PMC7080102

[43] Wosten-van Asperen RM, Lutter R, Specht PA, Moll GN, van Woensel JB, et al (2011) Acute respiratory distress syndrome leads to reduced ratio of ACE/ACE2 activities and is prevented by angiotensin-(1-7) or an angiotensin II receptor antagonist. J Pathol 225: 618–627. 22009550 10.1002/path.2987

[44] Nogueira-Silva C, Carvalho-Dias E, Piairo P, Nunes S, Baptista MJ, et al (2012) Local fetal lung renin-angiotensin system as a target to treat congenital diaphragmatic hernia. Mol Med 18: 231–243. 22113494 10.2119/molmed.2011.00210 PMC3320134

[45] Lasaitiene D, Chen Y, Nannmark U, Wollmer P, Friberg P (2004) Neonatal ACE inhibition in rats interferes with lung development. Clin Physiol Funct Imaging 24: 65–68. 14717750 10.1046/j.1475-0961.2003.00530.x

